# Cardiac function during weaning failure: the role of diastolic dysfunction

**DOI:** 10.1186/s13613-017-0348-4

**Published:** 2018-01-09

**Authors:** Ferran Roche-Campo, Alexandre Bedet, Emmanuel Vivier, Laurent Brochard, Armand Mekontso Dessap

**Affiliations:** 1 Service de Réanimation Médicale, DHU A-TVB, Hôpitaux Universitaires Henri Mondor, Assistance Publique – Hôpitaux de Paris, 51 Avenue du Maréchal de Lattre de Tassigny, 94010 Créteil Cedex, France; 2 Servei de Medicina Intensiva, Hospital Verge de la Cinta, Tortosa, Tarragona Spain; 3 Groupe de Recherche Clinique CARMAS, Institut Mondor de Recherche Biomédicale, Faculté de Médecine de Créteil, Université Paris Est Créteil, 94010 Créteil, France; 4 Service de Réanimation Polyvalente, Centre hospitalier Saint-Joseph Saint-Luc, Lyon, France; 5 Keenan Research Centre and Critical Care Department, St Michael’s Hospital, Toronto, Canada; 6 Interdepartmental Division of Critical Care Medicine, University of Toronto, Toronto, Canada

**Keywords:** Weaning, Diastolic function, Relaxation, Diastolic reserve

## Abstract

**Background:**

Cardiac dysfunction is a common cause of weaning failure. Weaning shares some similarities with a cardiac stress test and may challenge active phases of the cardiac cycle-like ventricular contractility and relaxation. This study aimed at assessing systolic and diastolic function during the weaning process and scrutinizing their dynamics during weaning trials.

**Methods:**

Echocardiography was performed during baseline ventilator settings to assess cardiac function at the initiation of the weaning process and at the start and the end of consecutive weaning trials (performed at day-1, day-2, and before extubation if applicable) to explore the evolution of left ventricle contractility and relaxation in a subset of patients.

**Results:**

Among 67 patients included, weaning was prolonged (≥ 7 days) in 18 (27%) patients and short (< 7 days) in 49 (73%). Prevalence of systolic dysfunction and isolated diastolic dysfunction before the initiation of weaning process were 37 and 17%, respectively. Isolated diastolic dysfunction was more frequent in patients with prolonged weaning as compared to their counterparts. Thirty-one patients were explored by echocardiography during consecutive weaning trials. An increase in filling pressures with an alteration of ventricular relaxation (as assessed by a decrease in tissue Doppler early mitral diastolic wave velocity) was found during failed weaning trials.

**Conclusions:**

Isolated diastolic dysfunction was associated with a prolongation of weaning. Increased filling pressures with left ventricle relaxation impairment may be a key mechanism of weaning trial failure.

**Electronic supplementary material:**

The online version of this article (10.1186/s13613-017-0348-4) contains supplementary material, which is available to authorized users.

## Background

Weaning from mechanical ventilation is an essential step in the care of critically ill intubated patients, accounting for approximately 40% of the total duration of mechanical ventilation [1]. Given that increased time on mechanical ventilation is associated with higher mortality rates [2], it is crucial to safely wean the patient from the ventilator as soon as possible. Pulmonary edema is one of the main causes of weaning failure [3], and cardiovascular dysfunction during weaning may involve systolic [4] and/or diastolic alterations [5, 6].

In healthy subjects, relaxation enhancement during exercise blunts the increase in venous return to maintain normal filling pressures [7]. However, an impaired relaxation may be unmasked during exercise in patients with mild symptoms of heart failure, irrespective of the presence of diastolic dysfunction at rest [8, 9]. Because weaning shares some similarities with a cardiorespiratory stress test [10, 11], the same pathophysiology is conceivable to explain the increase in filling pressures during weaning failure of cardiac origin. We hypothesized that diastolic dysfunction at baseline or impaired diastolic relaxation during weaning trials may mediate weaning failure.

The present study had two primary aims: first, to assess cardiac function at initiation of the weaning process and evaluate its association with weaning outcomes; second, to assess the dynamics of left ventricle (LV) contractility and relaxation in a subgroup of patients during consecutive weaning trials.

## Methods

### Study population

This ancillary study, planned a priori, was performed in one (Henri Mondor University hospital, Creteil, France) of the nine centers participating in the B-type natriuretic peptide (BNP) for the fluid Management of Weaning (BMW) trial [12]. The BMW study was a randomized, controlled trial comparing a biomarker-guided depletive fluid management strategy to usual care during ventilator weaning. A detailed description of the BMW study design (NCT00473148) has been published previously [12]. Inclusion criteria of the BMW study were those allowing early initiation of ventilator weaning in patients receiving mechanical ventilation for at least 24 h. Permanent non-inclusion criteria were: pregnancy or lactation, age < 18 years, known allergy to furosemide or sulfonamides, tracheostomy at inclusion, hepatic encephalopathy, cerebral edema, acute hydrocephalus, myasthenia gravis, acute idiopathic polyradiculoneuropathy, decision to withdraw life support, and prolonged cardiac arrest with a poor neurological prognosis. The protocol was approved by our institution’s local ethics committee (Comité de Protection des Personnes Ile-de-France IX, approval number 06–035), and informed consent was signed by the patient or a close relative. The main result of the BMW trial was to show that a BNP-driven depletive fluid management strategy decreased the duration of weaning without increasing adverse events [12].

### Study protocol

To standardize the weaning process, patients were ventilated using a computer-driven automated weaning system (AWS, Evita Smart Care System, Dräger Medical, Lubeck, Germany), which gradually decreased the pressure support level (while maintaining the patient within a zone of respiratory comfort), as previously described [13]. When the AWS declared the patient ready for separation, extubation was performed as soon as possible (including during the night), provided the patient met the other criteria required for extubation [12].

In a subgroup of 31 patients for whom echocardiography availability allowed consecutive examinations, a daily weaning trial was performed if the patient was still ventilated with the AWS and not ready for separation. The weaning trial lasted one hour and consisted of a low-pressure support trial (10 cm H_2_O in case of moisture humidifier or 7 cm H_2_O in case of heated humidifier) with zero-PEEP [11]. Criteria for weaning trial failure were: respiratory rate > 35 breaths/min and/or increased accessory muscle activity, SpO_2_ < 90%, heart rate > 140 beats/min, systolic blood pressure > 200 or < 80 mmHg, diaphoresis and clinical signs of distress. More information about the study protocol is available in the data supplement (Additional file 1: ESM Study protocol).

### Classification of weaning

Successful extubation was defined as patient alive and without reintubation 72 h after extubation. We adapted the WIND study classification of weaning process [2] to the use of the AWS and further summarized this classification into two groups as follows: short weaning (patients successfully extubated within 6 days of AWS) and prolonged weaning (patients still ventilated after 7 days of AWS or more). Patients who died between 1 to 6 days and after 6 days of AWS were classified as short and prolonged weaning, respectively. This dichotomization was driven by the need for parsimony as per the limited sample size, and the fact that prolonged weaning identifies a subgroup of patients at increased risk of mortality, as compared to their counterparts [14].

### Echocardiography

In all included patients (*n* = 67), echocardiography was performed to assess cardiac function during baseline ventilator settings (in pressure support ventilation), just before starting the weaning process with the AWS. In addition, we examined in a subset of patients (*n* = 31) whether weaning trials (low-pressure support with zero-PEEP) could induce an alteration of systolic or diastolic function, independently from their baseline function. In this subgroup, echocardiography was performed at the beginning and end of consecutive weaning trials performed at day-1, day-2, and before extubation. All echocardiographic examinations were performed by a single trained operator (FRC, with competence in advanced critical care echocardiography) not involved in patient care, using a transthoracic ultrasound device (EnVisor, Philips ultrasound, Bothell, WA). Briefly, the following echocardiographic views were examined with the patient in the semi-recumbent position: four-chamber and two-chamber long-axis views to assess left ventricle ejection fraction (LVEF), computed from LV volumes using the bi-plane Simpson method when image quality was suitable, or visually estimated when poor image quality did not allow sufficient identification of the endocardium; tissue Doppler peak systolic (*s*′) wave at the lateral mitral valve annulus; right ventricle size (a dilated right ventricle was defined by an end-diastolic right ventricle/left ventricle area ratio > 0.6) and function (using the tricuspid annular plane systolic excursion); diastolic function [using pulsed-wave Doppler early (*E*) and late (A) diastolic wave velocities at the mitral valve, and tissue Doppler early (*e*′) and late (*a*′) diastolic wave velocities at the lateral mitral valve annulus]. Systolic dysfunction was defined as LVEF < 50%. Isolated diastolic dysfunction (with preserved LVEF) was defined using the 2016 European Society of Cardiology guidelines (LVEF ≥ 50% with plasma BNP concentration > 35 pg/mL and [*E*/*e*′ ratio ≥ 13 or *e*′ < 9]) [15]. Because there is no single widely accepted definition for diastolic dysfunction, we also assessed, as a sensitivity analysis (available in Additional file 2: Table 1), other definitions proposed by scientific societies and experts, as follows: (1) LVEF ≥ 50% and *e*′ < 8 cm/s [16]; (2) LVEF ≥ 50% and (*E*/*e*′ ratio > 8 or *e*′/*a*′ ratio < 1) [17]; or (3) LVEF ≥ 50%, *E*/*e*′ ratio > 8 and plasma BNP concentration > 200 pg/mL [18]. Dynamics of LV contractility and relaxation during weaning trials were assessed using the *s*′ and *e*′ waves, respectively [19–22]. Pulsed-wave Doppler flows were obtained below the aortic valve to assess LV outflow tract for cardiac output computation. Mitral and aortic regurgitation were measured semi-quantitatively using color-flow Doppler and were considered severe at grades III–IV [23]. Echocardiographic images were digitally stored, and a computer-assisted evaluation was performed off-line by two trained operators (EV, AMD). All measures were averaged over a minimum of three cardiac cycles (five to ten in case of non-sinus rhythm).

### Statistical analysis

The data were analyzed using SPSS Base 20 (IBM-SPSS Inc, Chicago, IL, USA). Categorical variables were expressed as numbers (percentage) and continuous data as medians (25th–75th percentiles), unless otherwise specified. We used the Chi-squared or Fisher exact test to compare categorical variables between groups and the Student’s T test, Mann–Whitney test or Wilcoxon paired test to compare continuous variables, as appropriate. A *p* value of < 0.05 was considered statistically significant.

## Results

### Patient population, cardiac function and weaning outcome

**Fig. 1:**
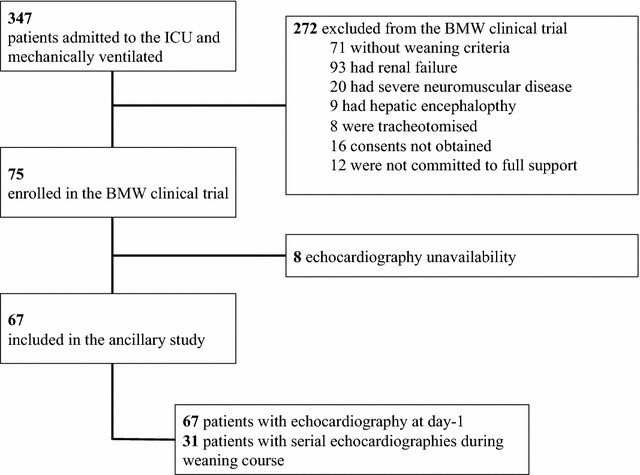
Study flow chart. *BMW* B-type natriuretic peptide for the fluid management of weaning

Among the 75 participants enrolled, we have explored cardiac function in 67. Eight patients were excluded because of echocardiography unavailability (Fig. 1). Weaning was prolonged in 18 (27%) patients and short in 49 (73%) patients. All patient characteristics were similar between groups, except for a lower PaO_2_/FiO_2_ ratio at inclusion in patients with prolonged weaning as compared to their counterparts (Table 1). Before starting the weaning process, the majority of patients had an impaired cardiac function; overall, the prevalence of systolic dysfunction and isolated diastolic function were 37 and 17%, respectively. Isolated diastolic dysfunction was more frequent in patients with prolonged weaning (≥ 7 days) as compared to their counterparts (Table 1). Tricuspid annular plane systolic excursion was also lower in patients with prolonged weaning as compared to others, while other echocardiographic variables were similar between groups (Table 1). End-diastolic right ventricle/left ventricle area ratio and pulmonary artery systolic pressure were similar in patients with or without isolated diastolic dysfunction: 0.59 [0.58–0.66] versus 0.56 [0.44–0.67], *p* = 0.46 and 43 [25–61] versus 37 [25–50] mmHg, *p* = 0.41, respectively. Cardiovascular treatments and weaning outcomes are reported in Table 2. Most patients received diuretics, including all those with prolonged weaning, but the latter group had a more positive fluid balance during weaning as compared to the short weaning group. In comparison with the short weaning group, fewer patients in the prolonged weaning group received vasodilators. Weaning duration, ICU length of stay and mortality were significantly greater in the prolonged weaning group (Table 2).

**Table 1:** Patient characteristics and echocardiographic variables just before the weaning process, according to weaning category (*n* = 67)

	All patients (*n* = 67)	Weaning		*p*
		Short (*n* = 49)	Prolonged (*n* = 18)	
**Patient characteristics**				
Age, year	64 (47–76)	61 (49–75)	69 (44–81)	0.50
Male sex	44 (66)	31 (63)	13 (72)	0.49
SAPS II at ICU admission	46 (34–53)	44 (34–58)	46 (34–50)	0.50
*Comorbidities*				
Hypertension	33 (49)	23 (47)	10 (56)	0.52
Diabetes	17 (26)	9 (19)	8 (44)	0.05
Chronic obstructive pulmonary disease	17 (25)	13 (27)	4 (22)	> 0.99
History of ischemic heart disease	13 (19)	11 (22)	2 (11)	0.48
Atrial fibrillation	22 (33)	13 (27)	9 (50)	0.07
*Reason for intubation*				0.67
Coma	13 (19)	9 (18)	4 (22)	
Septic shock	8 (12)	5 (10)	3 (17)	
Cardiogenic pulmonary edema	17 (25)	14 (29)	3 (17)	
Pneumonia	18 (27)	12 (25)	6 (33)	
Cardiac arrest	4 (6)	4 (8)	0 (0)	
Surgery	7 (10)	5 (10)	2 (11)	
*Events between ICU admission and inclusion*				
Septic shock	33 (49)	25 (51)	8 (44)	0.63
Ventilator-associated pneumonia	15 (22)	9 (18)	6 (33)	0.21
Acute respiratory distress syndrome	26 (39)	18 (37)	8 (44)	0.56
Use of neuromuscular blockers	11 (16)	9 (18)	2 (11)	0.71
Cumulative fluid balance before inclusion, mL	4322 (949–7898)	4322 (175–7253)	4228 (1757–17,957)	0.27
Duration of invasive MV before inclusion, days	3 (2–6)	3 (2–6)	5 (3–13)	0.08
*Clinical and biological data at inclusion*				
SOFA score	4 (3–6)	4 (3–6)	5 (4–6)	0.25
Systolic arterial pressure, mmHg	129 (122–144)	132 (113–146)	127 (108–136)	0.44
Heart rate, beats/min	93 (82–105)	93 (83–106)	91 (79–104)	0.60
Respiratory rate, beats/min	25 (19–30)	23 (18–29)	29 (27–33)	0.06
RPP, beats/min·mmHg	12,500 (11,288–15,346)	12,576 (10,744–15,520)	12,423 (12,245–13,035)	> 0.99
Arterial blood gases				
pH, units	7.44 (7.40–7.47)	7.44 (7.40–7.47)	7.44 (7.41–7.47)	0.77
PaO_2_/FiO_2_ ratio, mmHg	210 (182–270)	222 (188–277)	186 (153–226)	< 0.01
PaCO_2_, mmHg	41 (35–46)	40 (35–46)	42 (37–49)	0.42
BNP, pg/ml	331 (114–602)	302 (108–588)	415 (114–842)	0.51
Protidemia, g/L	58 (51–66)	58 (54–66)	54 (49–66)	0.39
Creatinine, micromol/L	79 (57–101)	81 (59–98)	73 (55–107)	0.96
Randomization in the interventional group	34 (51)	25 (51)	9 (50)	0.94
**Echocardiographic variables**				
LVEF, %	55 (40–60)	50 (37–60)	60 (50–62)	0.26
Cardiac index, L/min/m2	3.0 (2.2–3.6)	3.1 (2.3–3.6)	2.7 (2.1–3.7)	0.52
*Systolic dysfunction*				
LVEF < 50%	25 (37)	21 (43)	4 (22)	0.12
*Diastolic dysfunction* ^a^				
LVEF ≥ 50% and BNP > 35 pg/mL and (*E*/*e*′ ratio ≥ 13 or *e*′ < 9)	11 (17)	4 (8)	7 (39)	0.01
Heart valve disease^b^	23 (34)	14 (29)	9 (50)	0.10
RV/LV area ratio	0.6 (0.5–0.7)	0.6 (0.4–0.7)	0.6 (0.5–0.7)	0.67
Tricuspid annular plane systolic excursion, cm	1.9 (1.5–2.6)	2.1 (1.7–2.7)	1.5 (1.3–1.9)	0.03
Systolic pulmonary artery pressure, mmHg	38 (25–51)	37 (25–49)	46 (25–66)	0.16

*SAPS* Simplified Acute Physiologic Score, *ICU* intensive care unit, *MV* mechanical ventilation, *SOFA* sequential organ failure assessment, *RPP* product of heart rate and systolic arterial pressure, *FiO*_*2*_ fraction of inspired oxygen, *BNP* B-type natriuretic peptide, *LVEF* left ventricle ejection fraction, *E* early diastolic velocity measured using Doppler transmitral flow, *A* late diastolic velocity measured using Doppler transmitral flow, *e*′ early peak diastolic velocity of mitral annulus, *a*′ late peak diastolic velocity of mitral annulus, *RV* right ventricular end-diastolic area, *LV* left ventricular end-diastolic area

^a^Diastolic function could not be assessed in one patient for *e*′ and in two patients for *E*/*e*′ ratio

^b^Heart valve disease is defined as a severe aortic or mitral regurgitation (grade III/IV). Data are presented as *n* (%) or median (1st quartile–3rd quartile)

**Table 2:** Cardiovascular treatments and outcomes according to weaning category (*n* = 67)

	All patients (*n* = 67)	Weaning		*p*
		Short (*n* = 49)	Prolonged (*n* = 18)	
*Cardiovascular treatments during weaning*				
Diuretics	56 (84)	38 (78)	18 (100)	0.03
Dobutamine	19 (28)	15 (31)	4 (22)	0.50
Vasodilator	30 (45)	26 (53)	4 (22)	0.02
Amiodarone	19 (28)	13 (27)	6 (33)	0.58
Any cardiovascular treatment	62 (93)	44 (90)	18 (100)	0.31
Average daily furosemide dose during weaning, mg	25 (4–59)	30 (1–59)	22 (6–56)	0.94
Average daily fluid balance during weaning, mL	− 757 (− 2016 to − 81)	− 1202 (− 2342 to − 477)	51 (− 638 to 449)	< 0.01
Average daily urine output during weaning, mL	2588 (1971 to 3863)	2950 (2205 to 4167)	1967 (1649 to 2912)	0.02
*Outcomes*				
Time to first successful extubation, days	2 (1–6)	1 (1–2)	13 (10–32)	< 0.01
Ventilator free days at day-28, days	24 (18–27)	27 (25–27)	0 (0–16)	< 0.01
Time to discharge from ICU, days	9 (5–18)	7 (4–10)	31 (15–53)	< 0.01
Time to discharge from hospital, days	28 (15–53)	22 (13–32)	39 (22–53)	0.07
ICU mortality	9 (13)	2 (4)	7 (39)	< 0.01
Hospital mortality	10 (15)	3 (6)	7 (39)	< 0.01

Data are presented as *n* (%) or median (1st quartile–3rd quartile). Patient who died before day-28 had 0 ventilator free days

*ICU* intensive care unit

### Dynamics of LV contractility and relaxation during weaning trials

**Fig. 2:**
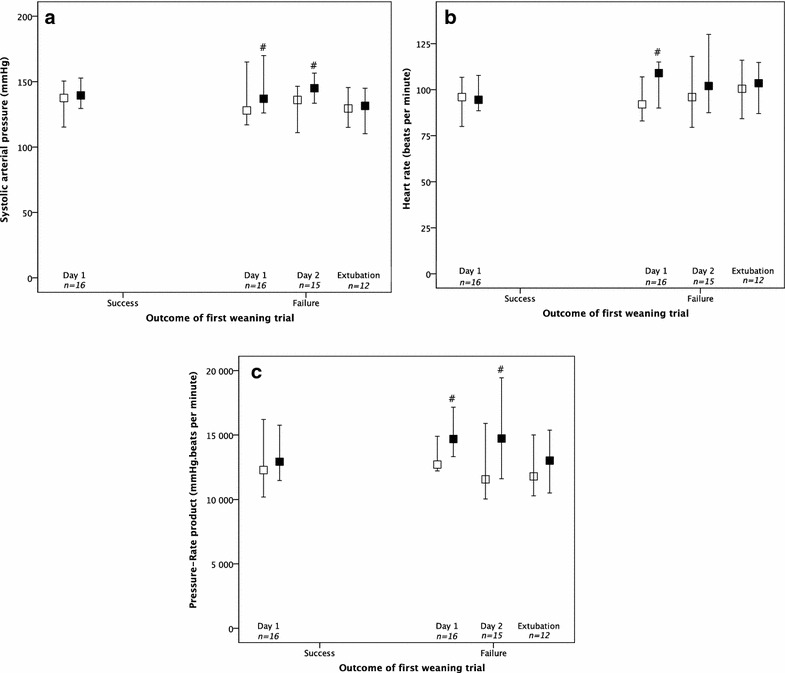
Systolic arterial pressure (**a**), heart rate (**b**) and pressure-rate product (**c**) at the start (white square) and the end (black square) of consecutive weaning trials during the weaning process (*n* = 31), according to first trial outcome (success or failure). ^#^*p* value < 0.05 as compared to the start of weaning trial (Wilcoxon test)

**Fig. 3:**
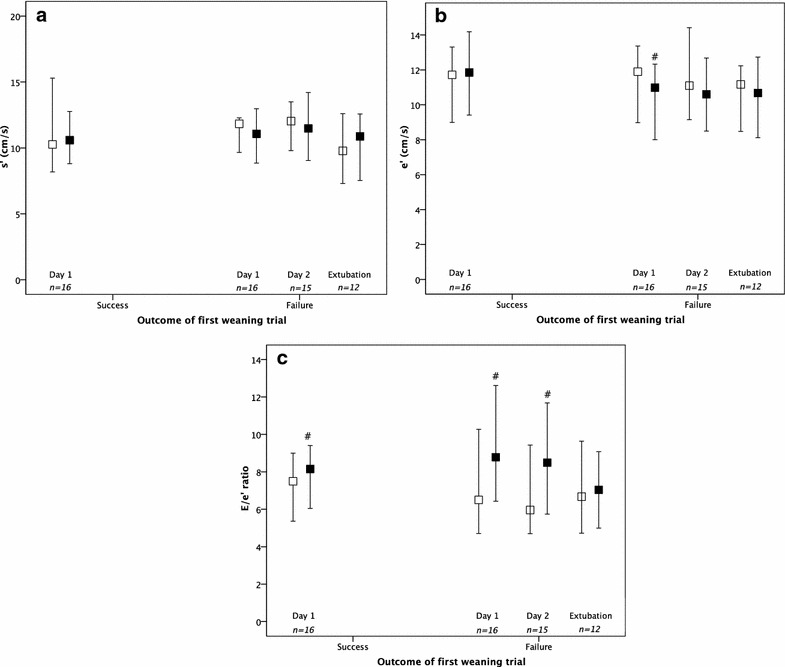
Tissue Doppler *s*′ wave (**a**), *e*′ wave (**b**) and *E*/*e*′ ratio (**c**), recorded with echocardiography at the start (white square) and the end (black square) of consecutive weaning trials during the weaning process (*n* = 31), according to first trial outcome (success or failure). ^#^*p* value < 0.05 as compared to the start of weaning trial (Wilcoxon test)

Among the 67 patients included, 31 were explored during consecutive weaning trials (Additional file 2: Table 2). Sixteen of these patients (52%) successfully passed the first weaning trial (day-1), whereas 15 (48%) failed. The evolution of cardiac clinical parameters and echocardiographic parameters during consecutive weaning trials (day-1, day-2, and before extubation) are displayed in Figs. 2 and 3, respectively. Failure of weaning trial was more often associated with an increase in systolic arterial pressure, heart rate and their product (pressure-rate product), as compared with weaning trial successes (Fig. 2). A marked increase in LV filling pressures (as assessed by *E*/*e*′ ratio) concomitant with an alteration of diastolic relaxation (as assessed by *e*′ velocity) were found in failed weaning trials (Fig. 3, Table 3). The *e*′ velocity increased in fewer (6.7%) and decreased in greater (93.3%) number of patients who failed weaning trials, as compared to successes (*p* < 0.001).

**Table 3:** Percent change in echocardiographic variables between the start and the end of the first weaning trial (*n* = 31), according to outcome (success or failure)

	All *n* = 31	Success *n* = 16	Failure *n* = 15	*p*
Delta *s*′^a^	− 3% (− 12%; 9%)	− 3% (− 7%; 5%)	− 2% (− 15%; 9%)	0.872
Delta *e*′	− 3% (− 12%; − 4%)	3% (− 9%; 6%)	− 6% (− 18%; − 3%)	0.02
Delta *E*	13% (8%; 19%)	12% (7%; 16%)	14% (11%; 23%)	0.20
Delta *E*/*e*′	16% (9%; 25%)	10% (4%; 14%)	26% (20%; 28%)	< 0.01

Data are presented as median (1st quartile; 3rd quartile)

*s*′ peak systolic velocity at the lateral mitral valve annulus, *e*′ early peak diastolic velocity of mitral annulus, *E* early diastolic velocity measured using Doppler transmitral flow

^a^*s*′ could not be assessed in three patients

## Discussion

We herein report a high prevalence of cardiac dysfunction at initiation of weaning. Prolonged weaning was associated with a predominantly isolated diastolic rather than systolic dysfunction in our cohort. Echocardiographic exploration suggested that LV relaxation impairment with increased filling pressures may be a key mechanism of failed weaning trials.

### Cardiac dysfunction before the initiation of weaning

Cardiac dysfunction plays a critical role in weaning outcome. In patients with prolonged weaning (≥ 7 days) in our series, systolic and isolated diastolic dysfunction were found in 22 and 39% of patients, respectively. Systolic dysfunction is a known risk factor for extubation failure [4]. However, in patients with preserved LVEF, increase in preload (volume status) and afterload (arterial stiffness) during weaning may also impair LV compliance and provoke pulmonary edema, especially in case of pre-existing diastolic dysfunction [24]. Our results are consistent with some previous reports describing diastolic dysfunction as a risk factor for weaning failure [5, 6, 25]. The heterogeneity of diastolic dysfunction definitions may explain the variability of its incidence and prevalence in critically ill patients [26].

### Cardiac dynamics during weaning

During weaning, removal of positive-pressure ventilation increases LV preload and afterload, inducing some physiologic changes similar to those observed during a cardiovascular stress test. Tachycardia and hypertension are two major determinants of diastolic dysfunction. They were more pronounced during weaning failure in our series and have been reported as frequent features of weaning-induced cardiac dysfunction [27]. Pressure-rate product was significantly increased during failed weaning trials, as compared to successes. Tachycardia could participate in the alteration of diastolic function by reducing diastolic filling time and/or decreasing coronary perfusion [28]. In addition, LV diastolic performance has been shown to be strongly influenced by the hypertensive response to exercise. Hypertension is well known to exacerbate heart failure in patients with preserved ejection fraction [29].

The fall in LV pressure during relaxation is a key determinant of diastolic function, and depends on intrinsic (contractility, LV stiffness) and extrinsic (preload, afterload) factors [30, 31]. The *E* wave velocity of mitral inflow assesses the early diastolic filling of LV, primarily reflecting the driving pressure between the left atrium and the left ventricle, and is therefore affected by preload and relaxation. The *e*′ velocity, measured with tissue Doppler at the lateral mitral valve annulus, is usually used to correct for the effect of LV relaxation on *E* wave [21, 22, 32]. Thus, the *E*/*e*′ ratio is considered a reliable measure of LV filling pressure, with minimal influence of intrinsic relaxation or age [33]. Although the assessment of diastolic function with these validated Doppler indices is usually highly reproducible [21, 32, 34], the detection of small changes may be challenging for non-experts in routine practice.

We found an increase in *E*/*e*′ ratio during weaning trial, which is compatible with an elevation of filling pressures, as previously demonstrated [25, 35]. Several studies have found an independent association between *e*′ and LV relaxation [22, 33, 36]. As compared to the *E* wave velocity, preload may have a minimal effect on *e*′ [21, 22, 37], especially in patients with diastolic dysfunction [38]. Our finding that *e*′ velocity tends to reduce during failed weaning trials is therefore compatible with an impaired diastolic relaxation in these patients, although a causality cannot be ascertained. This phenomenon is compatible with a lack of diastolic reserve, which may prevent the ability of LV to improve diastolic function and maintain normal filling pressures during stress [39–41]. Several studies evaluated the diastolic reserve with echocardiography in patients with heart failure and preserved ejection fraction [8, 9]. A decrease in *e*′ wave, together with a concomitant increase in *E*/*e*′ ratio, was the strongest markers of impaired diastolic reserve in these patients. Similar results were found in our study during failed weaning trials. A dynamic alteration of diastolic function during weaning stress in patients lacking diastolic reserve could be a possible mechanism of weaning failure, independently from the cardiac function at baseline. This hypothesis is in accordance with a previous work by Moschietto et al., who suggested the evolution of the LV relaxation rate during a spontaneous breathing trial (SBT) as the key factor in weaning outcome. However, the decrease in *e*′ velocity during failed weaning trials is in contrast with this former study which found no significant variation during SBT. This discrepancy may be explained by the timing of the second echocardiography. These authors repeated echocardiographic examination only 10 min after starting the weaning trial, whatever its total duration [25], whereas we rather assessed dynamic changes at the end of the weaning trial. The modality of weaning trial may also play a critical role [11].

### Therapeutic implications

The key mechanism of weaning failure did not seem to involve systolic dysfunction in our study, as also suggested by others [5, 25, 35]. Inotropic support could hypothetically exacerbate stress-induced diastolic dysfunction by increasing heart rate and/or myocardial oxygen demand. Dobutamine was even used as a stress test to diagnose heart failure with preserved ejection fraction [42]. Isolated diastolic dysfunction is frequent in ICU patients, especially in the elderly [43], and its diagnosis may deserve a specific therapeutic management in case of complicated weaning. Conservative and depletive fluid management are known to decrease the duration of ventilator support [44] and weaning [12], respectively. In our series, we could not assess the specific role of diuretics on SBT-induced cardiovascular burden because the vast majority of patients in the entire cohort received diuretics. Despite the use of diuretics in all patients with prolonged weaning, the urine output was lower and the fluid balance was higher in this group. The control of volume overload during diastolic heart failure may require higher doses of furosemide and/or the association of thiazide-like diuretics [29]; these strategies should be tested in future trials of fluid management during weaning. Fewer patients with prolonged weaning were treated with vasodilators as compared to those with short weaning. Vasodilators may be used to blunt the hypertensive response to weaning and expedite separation from the ventilator [45]. Future trials are needed to determine the optimal blood pressure target during ventilator weaning. Whether aerobic exercise training in ventilated patients could improve the diastolic reserve [46], ameliorate the tolerance of weaning trials and fasten the weaning process also needs to be explored in future studies.

### Strengths and limitations

Strengths of our study include its prospective design and the detailed cardiac assessment using echocardiography. In particular, our study comprehensively assessed diastolic function at weaning start and its dynamics during consecutive weaning trials. Limitations include the monocentric setting and the limited sample size, which precluded any multivariable analysis of factors associated with prolonged weaning. Also, only a minority of patients explored consecutively fulfilled our definition of diastolic dysfunction, preventing any evaluation of the relationship between diastolic dysfunction at baseline and relaxation dynamics during weaning trials. The lack of a single gold standard definition of diastolic dysfunction complicated the analysis of our data, inasmuch as there was some patient heterogeneity concerning the changes in diastolic indices. Last, the characterization of the cardiac origin of weaning failure with tools like the pulmonary artery catheter or cardiac biomarkers would have strengthened our findings.

## Conclusions

Isolated diastolic dysfunction is more frequent in patients with prolonged weaning (≥ 7 days), as compared to those with a shorter weaning. In addition, failure of weaning trial seems associated with an elevation of filling pressures mediated by a stress-induced impairment of diastolic relaxation, which is compatible with a lack of diastolic reserve. Documentation of diastolic dysfunction as a cause of weaning failure is critical, as it may require specific management (especially vasodilators to blunt the hypertensive response to the weaning cardiovascular stress).

## Additional files

**Additional file 1.** Study protocol (data supplement)

**Additional file 2: Table S1.** Prevalence of diastolic dysfunction using several definitions, according to weaning category (*n* = 67). **Table S2.** Patient characteristics and echocardiographic variables before starting the weaning process of patients explored during consecutive weaning trials (*n* = 31)

## Abbreviations

A: late diastolic velocity measured using pulsed-wave Doppler transmitral flow
*a*′: tissue Doppler late diastolic wave velocities at the lateral mitral valve annulus
AWS: automated weaning system
BNP: B-type natriuretic peptide
*E*: early diastolic velocity measured using pulsed-wave Doppler transmitral flow
*e*′: tissue Doppler early diastolic wave velocities at the lateral mitral valve annulus
ICU: intensive care unit
LV: left ventricle
LVEF: left ventricle ejection fraction
PEEP: positive end-expiratory pressure
*s*′: tissue Doppler peak systolic wave velocity at the lateral mitral valve annulus
SpO_2_: peripheral oxygen saturation
SBT: spontaneous breathing trial

## References

[1] Esteban A, Alia I, Ibañez J, Benito S, Tobin MJ (1994). Modes of mechanical ventilation and weaning: a National Survey of Spanish Hospitals. Chest.

[2] Béduneau G, Pham T, Schortgen F, Piquilloud L, Zogheib E, Jonas M (2016). Epidemiology of weaning outcome according to a new definition. The WIND study. Am J Respir Crit Care Med.

[3] Liu J, Shen F, Teboul J-L, Anguel N, Beurton A, Bezaz N (2016). Cardiac dysfunction induced by weaning from mechanical ventilation: incidence, risk factors, and effects of fluid removal. Crit Care.

[4] Thille AW, Boissier F, Ben Ghezala H, Razazi K, Mekontso-Dessap A, Brun-Buisson C (2015). Risk factors for and prediction by caregivers of extubation failure in ICU patients: a prospective study*. Crit Care Med.

[5] Papanikolaou J, Makris D, Saranteas T, Karakitsos D, Zintzaras E, Karabinis A (2011). New insights into weaning from mechanical ventilation: left ventricular diastolic dysfunction is a key player. Intensive Care Med.

[6] Konomi I, Tasoulis A, Kaltsi I, Karatzanos E, Vasileiadis I, Temperikidis P (2016). Left ventricular diastolic dysfunction–an independent risk factor for weaning failure from mechanical ventilation. Anaesth Intensive Care.

[7] Ha J-W, Lulic F, Bailey KR, Pellikka PA, Seward JB, Tajik AJ (2003). Effects of treadmill exercise on mitral inflow and annular velocities in healthy adults. Am J Cardiol.

[8] Burgess MI, Jenkins C, Sharman JE, Marwick TH (2006). Diastolic stress echocardiography: hemodynamic validation and clinical significance of estimation of ventricular filling pressure with exercise. J Am Coll Cardiol.

[9] Chattopadhyay S, Alamgir MF, Nikitin NP, Rigby AS, Clark AL, Cleland JGF (2010). Lack of diastolic reserve in patients with heart failure and normal ejection fractionclinical perspective. Circ Heart Fail.

[10] Pinsky MR (2000). Breathing as exercise: the cardiovascular response to weaning from mechanical ventilation. Intensive Care Med.

[11] Cabello B, Thille AW, Roche-Campo F, Brochard L, Gómez FJ, Mancebo J (2010). Physiological comparison of three spontaneous breathing trials in difficult-to-wean patients. Intensive Care Med.

[12] Mekontso Dessap A, Roche-Campo F, Kouatchet A, Tomicic V, Beduneau G, Sonneville R (2012). Natriuretic peptide–driven fluid management during ventilator weaning. Am J Respir Crit Care Med.

[13] Lellouche F, Mancebo J, Jolliet P, Roeseler J, Schortgen F, Dojat M (2006). A multicenter randomized trial of computer-driven protocolized weaning from mechanical ventilation. Am J Respir Crit Care Med.

[14] Peñuelas O, Frutos-Vivar F, Fernández C, Anzueto A, Epstein SK, Apezteguía C (2011). Characteristics and outcomes of ventilated patients according to time to liberation from mechanical ventilation. Am J Respir Crit Care Med.

[15] Ponikowski P, Voors AA, Anker SD, Bueno H, Cleland JGF, Coats AJS (2016). 2016 ESC Guidelines for the diagnosis and treatment of acute and chronic heart failure The Task Force for the diagnosis and treatment of acute and chronic heart failure of the European Society of Cardiology (ESC) Developed with the special contribution of the Heart Failure Association (HFA) of the ESC. Eur Heart J.

[16] Garcia MJ, Thomas JD, Klein AL (1998). New Doppler echocardiographic applications for the study of diastolic function. J Am Coll Cardiol.

[17] Kasner M, Westermann D, Steendijk P, Gaub R, Wilkenshoff U, Weitmann K (2007). Utility of Doppler Echocardiography And Tissue Doppler imaging in the estimation of diastolic function in heart failure with normal ejection fraction. Circulation.

[18] Paulus WJ, Tschöpe C, Sanderson JE, Rusconi C, Flachskampf FA, Rademakers FE (2007). How to diagnose diastolic heart failure: a consensus statement on the diagnosis of heart failure with normal left ventricular ejection fraction by the Heart Failure and Echocardiography Associations of the European Society of Cardiology. Eur Heart J.

[19] Nikitin NP, Loh PH, de Silva R, Ghosh J, Khaleva OY, Goode K (2006). Prognostic value of systolic mitral annular velocity measured with Doppler tissue imaging in patients with chronic heart failure caused by left ventricular systolic dysfunction. Heart.

[20] Seo J-S, Kim D-H, Kim W-J, Song J-M, Kang D-H, Song J-K (2010). Peak systolic velocity of mitral annular longitudinal movement measured by pulsed tissue Doppler imaging as an index of global left ventricular contractility. Am J Physiol Heart Circ Physiol.

[21] Nagueh SF, Middleton KJ, Kopelen HA, Zoghbi WA, Quiñones MA (1997). Doppler tissue imaging: a noninvasive technique for evaluation of left ventricular relaxation and estimation of filling pressures. J Am Coll Cardiol.

[22] Sohn D-W, Chai I-H, Lee D-J, Kim H-C, Kim H-S, Oh B-H (1997). Assessment of mitral annulus velocity by Doppler tissue imaging in the evaluation of left ventricular diastolic function. J Am Coll Cardiol.

[23] Dujardin KS, Enriquez-Sarano M, Bailey KR, Nishimura RA, Seward JB, Tajik AJ (1997). Grading of mitral regurgitation by quantitative Doppler echocardiography: calibration by left ventricular angiography in routine clinical practice. Circulation.

[24] Zapata L, Vera P, Roglan A, Gich I, Ordonez-Llanos J, Betbesé AJ (2011). B-type natriuretic peptides for prediction and diagnosis of weaning failure from cardiac origin. Intensive Care Med.

[25] Moschietto S, Doyen D, Grech L, Dellamonica J, Hyvernat H, Bernardin G (2012). Transthoracic echocardiography with Doppler tissue imaging predicts weaning failure from mechanical ventilation: evolution of the left ventricle relaxation rate during a spontaneous breathing trial is the key factor in weaning outcome. Crit Care.

[26] De Meirelles Almeida CA, Nedel WL, Morais VD, Boniatti MM, de Almeida-Filho OC (2016) Diastolic dysfunction as a predictor of weaning failure: a systematic review and meta-analysis. J Crit Care [Internet]. 2016 Apr 18 [cited 2016 Apr 18]. http://www.sciencedirect.com/science/article/pii/S0883944116000897. 10.1016/j.jcrc.2016.03.007 27067288

[27] Grasso S, Leone A, De Michele M, Anaclerio R, Cafarelli A, Ancona G (2007). Use of N-terminal pro-brain natriuretic peptide to detect acute cardiac dysfunction during weaning failure in difficult-to-wean patients with chronic obstructive pulmonary disease *. Crit Care Med.

[28] Selby DE, Palmer BM, LeWinter MM, Meyer M (2011). Tachycardia-induced diastolic dysfunction and resting tone in myocardium from patients with normal ejection fraction. J Am Coll Cardiol.

[29] Redfield MM (2016). Heart failure with preserved ejection fraction. N Engl J Med.

[30] Buda AJ, Pinsky MR, Ingels NBJ, Daughters GTI, Stinson EB, Alderman EL (1979). Effect of intrathoracic pressure on left ventricular performance. N Engl J Med.

[31] Westermann D, Kasner M, Steendijk P, Spillmann F, Riad A, Weitmann K (2008). Role of left ventricular stiffness in heart failure with normal ejection fraction. Circulation.

[32] Nagueh SF, Smiseth OA, Appleton CP, Byrd BF, Dokainish H, Edvardsen T (2016). Recommendations for the evaluation of left ventricular diastolic function by echocardiography: an update from the American Society of Echocardiography and the European Association of Cardiovascular Imaging. J Am Soc Echocardiogr.

[33] Ommen SR, Nishimura RA, Appleton CP, Miller FA, Oh JK, Redfield MM (2000). Clinical utility of Doppler echocardiography and tissue Doppler imaging in the estimation of left ventricular filling pressures: a comparative simultaneous Doppler-catheterization study. Circulation.

[34] Frikha Z, Girerd N, Huttin O, Courand PY, Bozec E, Olivier A, et al (2015) Reproducibility in echocardiographic assessment of diastolic function in a population based study (The STANISLAS Cohort Study). PLoS ONE [Internet] 8:10(4). https://www.ncbi.nlm.nih.gov/pmc/articles/PMC4390157/. 10.1371/journal.pone.0122336 PMC4390157 25853818

[35] Lamia B, Maizel J, Ochagavia A, Chemla D, Osman D, Richard C (2009). Echocardiographic diagnosis of pulmonary artery occlusion pressure elevation during weaning from mechanical ventilation*. Crit Care Med.

[36] Hasegawa H, Little WC, Ohno M, Brucks S, Morimoto A, Cheng H-J (2003). Diastolic mitral annular velocity during the development of heart failure. J Am Coll Cardiol.

[37] Graham RJ, Gelman JS, Donelan L, Mottram PM, Peverill RE (2003). Effect of preload reduction by haemodialysis on new indices of diastolic function 1979. Clin Sci Lond Engl.

[38] Nagueh SF, Sun H, Kopelen HA, Middleton KJ, Khoury DS (2001). Hemodynamic determinants of the mitral annulus diastolic velocities by tissue Doppler. J Am Coll Cardiol.

[39] Borlaug BA, Nishimura RA, Sorajja P, Lam CSP, Redfield MM (2010). Exercise hemodynamics enhance diagnosis of early heart failure with preserved ejection fractionclinical perspective. Circ Heart Fail.

[40] Borlaug BA, Jaber WA, Ommen SR, Lam CSP, Redfield MM, Nishimura RA (2011). Diastolic relaxation and compliance reserve during dynamic exercise in heart failure with preserved ejection fraction. Heart Br Card Soc.

[41] Holland DJ, Prasad SB, Marwick TH (2010). Contribution of exercise echocardiography to the diagnosis of heart failure with preserved ejection fraction (HFpEF). Heart.

[42] Erdei T, Smiseth OA, Marino P, Fraser AG (2014). A systematic review of diastolic stress tests in heart failure with preserved ejection fraction, with proposals from the EU-FP7 MEDIA study group. Eur J Heart Fail.

[43] Kitzman DW, Gardin JM, Gottdiener JS, Arnold A, Boineau R, Aurigemma G (2001). Importance of heart failure with preserved systolic function in patients ≥ 65 years of age. Am J Cardiol.

[44] The National Heart Lung, Network BIARDS (ARDS) CT (2006). Comparison of two fluid-management strategies in acute lung injury. N Engl J Med.

[45] Routsi C, Stanopoulos I, Zakynthinos E, Politis P, Papas V, Zervakis D (2010). Nitroglycerin can facilitate weaning of difficult-to-wean chronic obstructive pulmonary disease patients: a prospective interventional non-randomized study. Crit Care Lond Engl.

[46] Edelmann F, Gelbrich G, Düngen H-D, Fröhling S, Wachter R, Stahrenberg R (2011). Exercise training improves exercise capacity and diastolic function in patients with heart failure with preserved ejection fraction. J Am Coll Cardiol.

