# Supplementary material for: Cardiac function during weaning failure: the role of diastolic dysfunction
Source: Ann Intensive Care. 2018 Jan 9;8:2. doi: 10.1186/s13613-017-0348-4 (PMC5768586; doi:10.1186/s13613-017-0348-4)
Supplement: Supplementary file 1 — Additional file 1. Study protocol (data supplement) [file 13613_2017_348_MOESM1_ESM.docx]

**Additional file 1**

**METHODS**

**Study protocol**

This is an ancillary study of the BMW trial. In the BMW trial, patients ventilated in volume-assist or pressure-control mode were eligible for inclusion only if a pressure-support (PS) test was positive. The PS test (which is not a weaning trial) consisted in changing the ventilator mode to PS, without changing FiO2 or PEEP, as previously described (1). In patients already ventilated with PS at the time of inclusion, the positivity criteria of the PS test were checked. Patients fulfilling the inclusion and non-inclusion criteria and having a positive PS test were ventilated using the Smart Care system, starting with PS and PEEP levels similar to those used during the PS test. The protocol did not require performing a spontaneous breathing trial before enrolment. This inclusion strategy was reported in detail previously (2).

In this ancillary study of the BMW trial, all 67 patients were included as per the above-mentioned strategy and one echocardiography was performed to assess cardiac function during baseline ventilator settings (in pressure support ventilation), just before starting the weaning process with AWS (SmartCare). In addition, in a subgroup of patients (n=31), daily weaning trials (with minimal PS and no PEEP) were performed if they were still ventilated with the SmartCare system. In this subgroup, an echocardiography was performed at the beginning and end of consecutive weaning trials.

**REFERENCES**

1. Lellouche F, Mancebo J, Jolliet P, Roeseler J, Schortgen F, Dojat M, et al. A Multicenter Randomized Trial of Computer-driven Protocolized Weaning from Mechanical Ventilation. Am J Respir Crit Care Med. 2006 Oct 15;174(8):894–900.

2. Mekontso Dessap A, Roche-Campo F, Kouatchet A, Tomicic V, Beduneau G, Sonneville R, et al. Natriuretic Peptide–driven Fluid Management during Ventilator Weaning. Am J Respir Crit Care Med. 2012 Dec 15;186(12):1256–63.
