# Supplementary material for: Cardiac function during weaning failure: the role of diastolic dysfunction
Source: Ann Intensive Care. 2018 Jan 9;8:2. doi: 10.1186/s13613-017-0348-4 (PMC5768586; doi:10.1186/s13613-017-0348-4)
Supplement: Supplementary file 2 — Additional file 2: Table S1. Prevalence of diastolic dysfunction using several definitions, according to weaning category (n = 67). Table S2. Patient characteristics and echocardiographic variables before starting the weaning process of patients explored during consecutive weaning trials (n = 31) [file 13613_2017_348_MOESM2_ESM.docx]

**Table S1. Prevalence of diastolic dysfunction using several definitions, according to weaning category (n=67)**

|  | All patients  (n=67) | Weaning | | *p* |
| --- | --- | --- | --- | --- |
|  |  | Short  (n=49) | Prolonged  (n=18) |  |
| *Diastolic dysfunction^£^* |  |  |  |  |
| LVEF ≥50% and e’ <8 cm/s | 7 (11) | 2 (4) | 5 (28) | 0.01 |
| LVEF ≥50% and (E/e’ ratio >8 or e’/a’ ratio <1) | 24 (36) | 14 (29) | 10 (56) | 0.04 |
| LVEF ≥50% and E/e’ ratio >8 and BNP >200 pg/mL | 10 (15) | 4 (8) | 6 (35) | 0.01 |

*Definition of abbreviations: BNP=B-type natriuretic peptide. LVEF=left ventricle ejection fraction; E=early diastolic velocity measured using Doppler transmitral flow; A=late diastolic velocity measured using Doppler transmitral flow; e’=early peak diastolic velocity of mitral annulus; a’=late peak diastolic velocity of mitral annulus;. ^£^Diastolic function could not be assessed in one patient for e’ and in two patients for E/e’ ratio. Data are presented as n (%).*

**Table S2. Patient characteristics and echocardiographic variables before starting the weaning process of patients explored during consecutive weaning trials (n=31)**

|  | All patients  (n=31) |
| --- | --- |
| **Patient characteristics** |  |
| Age, year | 63 (52-74) |
| Male sex | 21 (67.7) |
| SAPS II at ICU admission | 35 (45-54) |
| *Comorbidities* |  |
| Hypertension | 15 (48.4) |
| Diabetes | 7 (23.3) |
| Chronic obstructive pulmonary disease | 6 (19.4) |
| History of ischemic heart disease | 6 (19.4) |
| Atrial fibrillation | 6 (19.4) |
| **Echocardiographic variables** |  |
| *Systolic dysfunction* |  |
| LVEF <50% | 13 (41.9) |
| LVEF <50% or need for inotrope to achieve LVEF ≥50% | 17 (54.8) |
| *Diastolic dysfunction^£^* |  |
| LVEF ≥50% and e’ <8 cm/s | 0 (0) |
| LVEF ≥50% and (E/e’ ratio >8 or e’/a’ ratio <1) | 10 (32.3) |
| LVEF ≥50% and E/e’ ratio >8 and BNP >200 pg/mL | 3 (9.7) |
| LVEF ≥50% and BNP >35 pg/mL and (E/e' ratio ≥13 or e' <9) | 3 (9.7) |

*Definition of abbreviations: SAPS=Simplified Acute Physiologic Score; ICU=intensive care unit; LVEF=left ventricle ejection fraction; E=early diastolic velocity measured using Doppler transmitral flow; A=late diastolic velocity measured using Doppler transmitral flow; e’=early peak diastolic velocity of mitral annulus; a’=late peak diastolic velocity of mitral annulus. Data are presented as n (%) or median (1^st^ quartile-3^rd^ quartile).*
